# Fungal endophytes associated with *Viola odorata* Linn. as bioresource for pancreatic lipase inhibitors

**DOI:** 10.1186/s12906-017-1893-y

**Published:** 2017-08-03

**Authors:** M. Katoch, A. Paul, G. Singh, S. N. C. Sridhar

**Affiliations:** 1 Department of Microbial Biotechnology, Indian Institute of Integrative Medicine, Canal Road, Jammu, 180001 India; 2 Laboratory of Natural Drugs, Department of Pharmacy, Birla Institute of Technology and Science Pilani (Pilani campus), Pilani, Rajasthan India

**Keywords:** Endophytic fungi, *Viola odorata*, Obesity, Pancreatic lipase inhibitory activity, Orlistat

## Abstract

**Background:**

As per the recent statistical reports of World Health Organisation (WHO), 13% of total global population is obese. Orlistat remains to be the only drug approved for the long term treatment of obesity. Recent findings highlighted severe adverse effects of orlistat that included hepatotoxicity, gall stones, kidney stones and acute pancreatitis. Therefore, search for new drug is required. The investigations based on endophytic natural products would prove pivotal in the global fight against this health issue.

**Methods:**

Obesity is associated with lipid metabolism involving pancreatic lipase enzyme. The inhibition of pancreatic lipase is demonstrated by using the extracts of endophytes isolated from *Viola odorata* Linn. In addition, endophytes were identified using ITS based rDNA sequencing.

**Results:**

Present study involves the isolation and identification of 27 endophytes from *V. odorata.* All the endophytes were evaluated for lipase inhibitory activities. The extracts of seven endophytes exhibited lipase inhibitory activity with IC_50_ < 10 μg/mL. The extract of VOLF4 (*Aspergillus* sp.) displayed promising lipase inhibitory activity (IC_50_ 3.8 μg/mL).

**Conclusion:**

The present study demonstrates that *V. odorata* harbors endophytic community with potent lipase inhibitory activity. VOLF4 is the potential endophyte. The extract of VOLF4 can be used to develop the potential drug to treat obesity.

## Background

Endophytes are microbes that reside in healthy plant tissue without causing any symptom to them [1]. They exhibit a variety of interactions with host varying from symbiotic to antagonistic thus affecting the plant growth, metabolism, ecology, fitness and evolution. These endophytes shape the plant community and profoundly influence the community structure and diversity of associated organisms i.e. bacteria, nematodes and insects [2–4]. Endophytes are involved in plant biology and have been found beneficial to environment and human beings. Endophytes are major contributors in the drug discovery and development process because they produce natural products which have diverse novel chemical structures and biological activities [5–10].

Obesity is a multifactorial metabolic disorder, which is characterized by an abnormal or excessive accumulation of lipids causing risk to human health ([11], http://www.who.int/topics/obesity/en). Statistics from reports of World Health Organisation (WHO) on obesity have projected a rapid growth of obese population. Almost 600 million adults are obese worldwide, accounting for 13% of total global population (http://www.who.int/mediacentre/factsheets/fs311/en). Further, obesity is associated with various comorbid conditions including insulin resistance, diabetes mellitus, cardiovascular diseases and certain cancers, that poses major health problem to the obese patients [12]. With an estimated 2.8 million deaths per year, obesity is the fifth leading risk of global deaths (http://easo.org/education-portal/obesity-facts-figures).

There are several targets that have been explored to treat or prevent the obesity. The pancreatic lipase (PL) is considered to be a successful and valid target due to its tolerable side effects [13–20]. The human PL (EC 3.1.1.3) is a primary digestive enzyme secreted from the exocrine glands of pancreas and is primarily involved in the hydrolysis of ester bonds of triglycerides [21]. The hydrolysis of the triglyceride esters by PL is represented by a series of events, initiated by an interfacial activation by the hydrophobic alkyl chains of the triglycerides, resulting in the open lid conformation followed by the nucleophilic attack of Ser 152 on to the carbonyl carbon of the ester linkage of the triglycerides [22, 23].

Orlistat (1), a PL inhibitor, remains to be the only drug approved for long term treatment of obesity (http://www.fda.gov/Drugs/DrugSafety/PostmarketDrugSafetyInformationforPatientsandProviders/ucm180076.htm). It is a saturated derivative of lipstatin, a potential natural PL inhibitor, produced from the actinobacterium *Streptomyces toxytricini* [24]. Recently, it has been reported that long term administration of orlistat (1) exhibits the severe adverse effects including hepatotoxicity, gall stones, kidney stones and acute pancreatitis (https://medlineplus.gov/druginfo/meds/a601244.html). The revised label for orlistat (1) with reference to cases of severe liver injury was approved by US FDA in 2010 for medication (http://www.fda.gov/Drugs/DrugSafety/PostmarketDrugSafetyInformationforPatientsandProviders/ucm213038.htm). Therefore, there is a requirement of developing safe and effective drugs for the treatment of obesity.
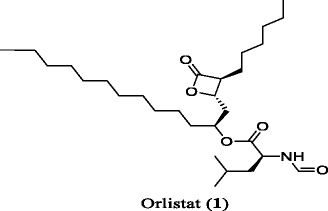


Investigations based on endophytic natural products would prove pivotal in the development of drug against such kind of health issues [25]. The search for safer and novel drugs based on natural product is under progress. The success can be achieved by selecting the underexplored and/or unexplored biological resources. North-Western Himalayas are blessed with unexplored plant and microbial biodiversity. Biological diversity implies chemical diversity with constant metabolic and environmental interactions continuously occurring and existing in ecosystems [26, 27]. The biological diversity possesses an unimaginable potential for chemical innovations and their potential use in the field of drug discovery [28, 29]. The endophytes belonging to unique environment and unique host are expected to be the producer of new chemical entities/natural products [30–32]. First time, Gupta et al. [33, 34] explored the fungal endophytes for lipase inhibitors.

The plant *Viola odorata* belongs to the family Violaceae and commonly known as sweet violet. The vernacular name is *Banafsha.* It is a hard and herbaceous flowering plant with medicinal properties and native to Europe, Asia, North America and Australia. In India, it grows in Kashmir and other parts of Western Himalayas in altitudes ranging from 1500 to 1800 m. It grows wild particularly in places exposed to direct sun light [35]. Since antiquity, it has been used in traditional medicine for curing a variety of respiratory ailments such as common cold, congestion, coughing and sore throat, insomnia, skin disorders, cancer pain, anxiety, lower blood pressure and headache [36–42]. Active ingredients of *V. odorata* are alkaloids, tannins, phenolics, coumarins, flavonoids, glycoside, saponins, methyl saicylate, mucilage, vitamin C and cyclotides [40, 43]. The plant has been reported to possess antibacterial, antifungal, antioxidant, antitumor, diuretic, laxative, analgesic, antihypertensive, antidyslipidemic, anti-inflammatory, antipyretic, sedative, anthelmintic and mosquito repellant properties [44–54].

Herein, we report the isolation and identification of endophytes from *Viola odorata* together with the investigation of lipase inhibitory activity of the extracts of endophytes.

## Methods

### Collection, identification and authentication of plant material

The matured plants of *V. odorata* were collected during March–April, 2014 randomly from Bhaderwah, Jammu and Kashmir (altitude of about 32.98°N 75.71°E), India. The species were identified taxonomically based on leaf and flower morphology and preserved in the herbarium (Accession No 23063). These plants were stored in an icebox and brought to the laboratory for further studies.

### Isolation of Endophytes

The endophytic fungi were isolated from *V. odorata* as per the method described by Strobel and Daisy [1] with slight modifications. Different tissues (roots, leaf nodes and leaves) of the disease free plants were carefully excised with a sterile scalpel. In the first instance, these tissues were cleaned by thorough washing in running tap water, followed by deionized (DI) water. Clean tissue pieces were sterilized in a series of solution: 70% ethanol; 1.0% sodium hypochlorite (*v*/v); 70% ethanol for 1 min in each solution. Finally, they were rinsed twice with sterile distilled water. After surface sterilization, tissues were dried on blotting sheets and cut into 0.5 cm^2^ pieces. These sterile small pieces were placed on water and potato dextrose agar (PDA) plates containing streptomycin (250 μg/mL) to inhibit the bacterial growth. The plates were wrapped with parafilm, incubated at 25 ± 2 °C and observed daily. The fringes of fungal mycelia growing from the tissues were subcultured on fresh PDA plates. The endophytic fungal isolates so obtained, were coded as per their tissue origin (VOR1, VOR3 from roots, VOLN1, VOLN2 from Leaf node and VOLF1, VOLF2, VOLF3 etc. from leaves)*.* These endophytes were stored in paraffin oil at 4 °C and were deposited in RN Chopra, Microbial Repository, IIIM.

### Identification of endophytes

Fungal endophytic isolates were finally identified by ITS based rDNA sequencing. Genomic DNA of the endophytes was extracted from the in vitro grown biomass of endophytes using the protocol described by Reader and Broda [55]. Approximately 1 g of dried mycelia was kept in liquid nitrogen and crushed into a fine powder. It was transferred to 10 mL of extraction buffer and vortexed thoroughly. The samples were incubated in water bath at 65 °C for 30 min with intermittent mixing. The tubes were centrifuged at 10,000 *g* for 5–10 min followed by extraction of aqueous layer with chloroform: isoamyl alcohol (24:1). Aqueous layer was collected and DNA was precipitated with 2.5–3 V of absolute ethanol in presence of 1/10th volume of sodium acetate. Tubes were inverted slowly to mix the contents and centrifuged at 8000 *g* for 20 min at 4 °C. White/transparent pellets thus obtained were washed with ice cold 70% ethanol followed by air drying. Dried pellets were dissolved in 20 μL of water (molecular biology grade). ITS sequences containing ITS1–5.8S–ITS2 region spanning 500–600 bp were amplified with ITS1 and ITS4 universal primers [56]. PCR reaction was set up in 50 μL containing DNA (1–10 ng), 1× PCR buffer (with 15 mM MgCl_2_), each dNTP (200 mM), each primer (10 pmol, IDT, Belgium) and 1 U *Taq* DNA polymerase (Promega, US). Cycling parameters were 5 min at 94 °C followed by 30 cycles of 94 °C for 30 s, 55 °C for 1 min, 72 °C for 1 min and a final extension for 10 min at 72 °C. The PCR product (10 μL) was resolved using agarose gel electrophoresis at 80 V. The amplified product was purified using a Gel Extraction Kit (Qiagen, USA) and sequencing reaction was set up in a 10 μL: 40–60 ng of purified PCR product, 3.2 pmol forward/reserve primer, Big Dye Terminator sequencing mix 8 μL (v. 3.1, Applied Biosystems). Samples were sequenced on an automated sequencing system (Applied Biosystems). Resultant sequences (KX621956-KX621982) were submitted to a gene bank and were blasted against the nucleotide database using blastn Tool of the US National Centre for Biotechnology Information (NCBI) for final identification of endophytes [57].

### Fermentation and extraction

For the extraction of biomolecules, the endophytic fungal isolates (twenty-seven) were cultured in 1 L Erlenmeyer flask containing 400 mL of potato dextrose broth (PDB) at 27 ± 2 °C for 10 days at 180 rpm (New Brunswick, USA). A 5 mm mycelial plug of 10-day old culture was used as inoculum. After 10 days, fermented culture of each endophyte was blended thoroughly in 20% methanol. Homogenate was extracted with one volume of methylene chloride (DCM) (HPLC grade). The extraction process was repeated four times. Solvent containing extract was striped off in a rotary evaporator leaving behind the solid powder, termed the crude extract. The stock solutions of extracts (10 mg/mL) were prepared in dimethyl sulfoxide (DMSO) and were used to evaluate the anti-obesity potential.

### Anti-obesity assay

Orlistat (**1)**, Porcine pancreatic lipase (Type II) and 4-nitrophenyl butyrate used for PL inhibition assay were procured from Sigma-Aldrich. Tris buffer and Sodium chloride (Sisco Research Laboratories) used for assay were of molecular biology grade. All other chemicals and solvents (analytical grade) were used without further purification.

PL inhibition assay was performed using the reported protocol [58], which was previously optimized in our laboratory [59]. Briefly, 50 mg of porcine pancreatic lipase was suspended in 10 mL of Tris–HCl buffer (containing 2.5 mmol of Tris and 2.5 mmol of NaCl, adjusted to pH 7.4 with HCl). The solution was subjected to vigorous shaking for 15 min, followed by centrifugation (4000 rpm, 291 K for 10 min). The supernatant was collected and used afresh as the enzyme solution.

Stock solutions of the extracts and orlistat were prepared in DMSO at linear concentrations ranging from 1.56–2000 μg/mL and 0.78–1000 μg/mL, respectively. The final reaction mixture comprised of 875 μL of buffer, 100 μL of enzyme and 20 μL of the compounds of various stock concentrations, pre-incubated for 5 min at 37 °C, followed by addition of 10 μL of the substrate (4-nitrophenyl butyrate, 10 mM in acetonitrile). The amount of DMSO in the final concentration did not exceed 2%. The absorbance of the final mixture was taken in microplate reader (EPOCH, BioTek) after 5 min at absorbance maxima of 4-nitrophenol (405 nm). The assay was performed in triplicate and the percentage inhibition was calculated using the formula

$$ \%\mathrm{Inhibition}=\left[\left(\left({\mathrm{A}}_{\mathrm{E}}-{\mathrm{A}}_{\mathrm{T}}\right)\right)/ {\mathrm{A}}_{\mathrm{E}}\right]\ \mathrm{X}\ 100 $$

Where, A_E_ is the absorbance of enzyme control (without inhibitor), and A_T_ is the difference between the absorbance of test sample with and without substrate. The IC_50_ of the compounds was calculated by plotting linear regression curve, and was compared to that of orlistat (reference standard). Porcine pancreatic lipase exhibited a K_m_ value of 92.36 μM and V_max_ of 0.065 μM/min [59].

### Statistical analysis

The inhibitory activity of endophytes against pancreatic lipase was examined with ANOVA and TUKEYS post hoc analysis using Graph Pad Prism software.

### Characterization of bioactive endophytes

The endophytic fungi that were exhibiting inhibitory activity against pancreatic lipase were characterized on the basis of the morpho-cultural and molecular taxonomy and phylogenetic characteristics.

For phylogenetic evaluation, endophytic ITS DNA sequence and downloaded sequences of their nearest neighbors were aligned in Alignment Explorer of MEGA4 software [60] using Clustal W option. Trimming and verification of the sequence alignment were carried out using the MUSCLE (UPGMA) algorithm. The Maximum Composite Likelihood and Neighbor-Joining methods were used to compute the evolutionary distances and history respectively. The robustness of the tree was assessed by bootstrap analysis with 1000 replications.

## Results

### Identification and characterization of the endophytic fungi

Endophytic fungi were isolated from healthy and symptomless tissues of *V. odorata* to assess their anti-obesity potential and were identified on the basis of morpho-cultural and microscopic characteristics. Further confirmation was made on the basis of their molecular identification carried out by ITS based rDNA sequence analysis. Details of the fungal endophytes, their isolation source, GenBank accession numbers, and closest sequence homolog are given in Table 1.

**Table 1:** Fungal endophytes isolated from *Viola odorata* L.

S No.	Endophyte	EMBL-Bank accession number	Most closely related strain (accession number)	Maximum Identity (%)
1	VOR1	KX621956	*Nigrospora* sp. AB693920	99
2	VOR3	KX621957	*Fusarium nematophilum* KF498859	99
3	VOR4	KX621958	*Penicillium* sp. *HZ-3* EU301633	99
4	VOR5	KX621959	*Fusarium* sp. AY729060	99
5	VOR6	KX621960	*Fusarium solani* KM235740	99
6	VOR7	KX621961	*Colletotrichum destructivum* KP259876	100
7	VOR10	KX621962	*Nectria haematococca* JX868649	100
8	VOR11	KX621963	*Colletotrichum truncatum* KU498294	99
9	VOR12	KX621964	*Colletotrichum destructivum* HQ674658	99
10	VOR14	KX621965	*Aspergillus awamori* LC106113	100
11	VOR15	KX621966	*Fusarium* sp. KF472154	99
12	VOR16	KX621967	*Fusarium oxysporum* KU872849	99
13	VOR17	KX621968	*Paecilomyces tenuis* GQ414523	99
14	VOR18	KX621969	*Fusarium nematophilum* KF577885	100
15	VOLN1	KX621970	*Aspergillus* sp. *JX164075*	99
16	VOLN2	KX621971	*Daldinia eschscholtzii* KJ466979	99
17	VOLN3	KX621972	*Aspergillus niger* EU440778	100
18	VOLN4	KX621973	*Colletotrichum destructivum* GU935874	99
19	VOLN5	KX621974	*Colletotrichum siamense* KP703352	99
20	VOLN7	KX621975	*Penicillium* sp. KC871048	99
21	VOLF1	KX621976	*Penicillium* sp. KC871048	99
22	VOLF2	KX621977	*Colletotrichum trifolii* AJ301942	99
23	VOLF3	KX621978	*Colletotrichum destructivum* KP259876	100
24	VOLF4	KX621979	*Aspergillus* sp. JX164075	99
25	VOLF5	KX621980	*Peniophora* sp. KF541333	99
26	VOLF6	KX621981	*Aspergillus japonicus* KC128815	99
27	VOLF7	KX621982	*Cladosporium tenuissimum* KP701910	100

The isolated endophytic fungi belonged to 11 different genera (Table 1). Most of the endophytic fungi belonged to Ascomycota phylum, except VOLF5 (*Peniophora sp*), which belonged to Basidiomycota phylum. *Colletotrichum* spp. (25.9%) showed the highest isolation frequency followed by *Fusarium* spp. (22.2%). Endophytes specifically isolated from the leaf tissues were *Colletotrichum trifolii, Cladosporium tenuissimum, Aspergillus japonicus* and *Peniophora sp.,* whereas *Daldinia eschscholtzii, Aspergillus niger* and *Colletotrichum siamense* were found specific to leaf nodes and roots were found to harbor *Nigrospora sp*., *Fusarium nematophilum, Fusarium solani, Colletotrichum truncatum, Nectria haematococca, Aspergillus awamori, Fusarium sp., Fusarium oxysporum,* and *Paecilomyces tenuis.*

### Anti-obesity activity

DCM extracts of the endophytes from *V. odorata* were subjected to PL inhibitory assay against porcine pancreatic lipase (Type II) using 4-nitrophenyl butyrate as substrate. Orlistat was used as reference standard and exhibited a very potent PL inhibition (IC_50_ of 0.49 μg/mL). Approximately 61% extracts showed potent lipase inhibitory activity with IC_50_ < 20 μg/mL, chosen as an acceptable limit (Table 2). Out of these 61% extracts, 7 endophytic extracts showed lipase inhibitory activity with IC_50_ < 10 μg/mL. Rest of nine extracts displayed moderate activity with IC_50_ 10–20 μg/mL. Further, VOLF4 (*Aspergillus* sp.) exhibited most potent PL inhibitory activity with an IC_50_ of 3.80 μg/mL, followed by VOLF5 (*Peniophora* sp*.*) and VOR5 (*Fusarium nematophilum*) with IC_50_ of 5.85 and 6.52 μg/mL, respectively**.** These results suggested that these extracts possess bioactive compounds with potential PL inhibitory activity. Furthermore, an analysis of the identified species against their IC_50_ values indicated that *Aspergillus* spp. along with most of the species of *Fusarium*, *Penicillium* and *Colletotrichum* exhibited good PL inhibitory activity.

**Table 2:** Anti-obesity activity (IC_50_ μg/mL) of endophytic fungi isolated from *V. odorata*

Culture code	Identified culture	Anti-obesity activity IC_50_ (μg/mL)
*Orlistat*	*-*	*0.49 ± 0.06*
VOR1^a,b,c,d,e,f^	*Nigrospora sp.*	63.81 ± 1.81
VOR3	*Fusarium nematophilum*	*18.55 ± 2.32*
VOR4	*Penicillium* sp.	*20.35 ± 1.47*
VOR5^c^	*Fusarium nematophilum*	*06.52 ± 0.95*
VOR6^d^	*Fusarium solani*	*07.00 ± 0.39*
VOR7	*Colletotrichum destructivum*	*26.81 ± 2.52*
VOR10^g^	*Nectria haematococca*	61.03 ± 3.72
VOR11	*Colletotrichum truncatum*	*18.57 ± 3.52*
VOR12	*Colletotrichum destructivum*	35.61 ± 2.18
VOR14	*Aspergillus awamori*	*13.83 ± 2.13*
VOR15^e^	*Fusarium* sp.	*07.32 ± 0.96*
VOR16	*Fusarium oxysporum*	30.72 ± 3.60
VOR17	*Paecilomyces tenuis*	*27.29 ± 1.88*
VOR18	*Fusarium nematophilum*	*10.82 ± 1.39*
VOLN1	*Aspergillus* sp.	*20.08 ± 2.02*
VOLN2	*Daldinia eschscholtzii*	*23.99 ± 1.84*
VOLN3	*Aspergillus niger*	*09.07 ± 0.86*
VOLN4	*Colletotrichum destructivum*	*11.65 ± 1.07*
VOLN5^f^	*Colletotrichum siamense*	*08.12 ± 1.80*
VOLN7	*Penicillium* sp.	*-*
VOLF1	*Penicillium* sp.	*11.25 ± 1.19*
VOLF2	*Colletotrichum trifolii*	*11.15 ± 3.15*
VOLF3	*Colletotrichum destructivum*	*24.78 ± 2.41*
VOLF4^a,g^	*Aspergillus* sp.	*03.80 ± 0.23*
VOLF5^b^	*Peniophora* sp.	*05.85 ± 0.5*
VOLF6	*Aspergillus japonicus*	*16.88 ± 1.15*
VOLF7	*Cladosporium tenuissimum*	32.07 ± 2.15

Same letters as superscript depicts significant difference at *P* < 0.05 in IC_50_ values of two endophytes _50_ values <30 µg/mL

Values in italics means IC_50_ <30 μg/mL

### Characterization of bioactive endophytes

**Fig. 1:**
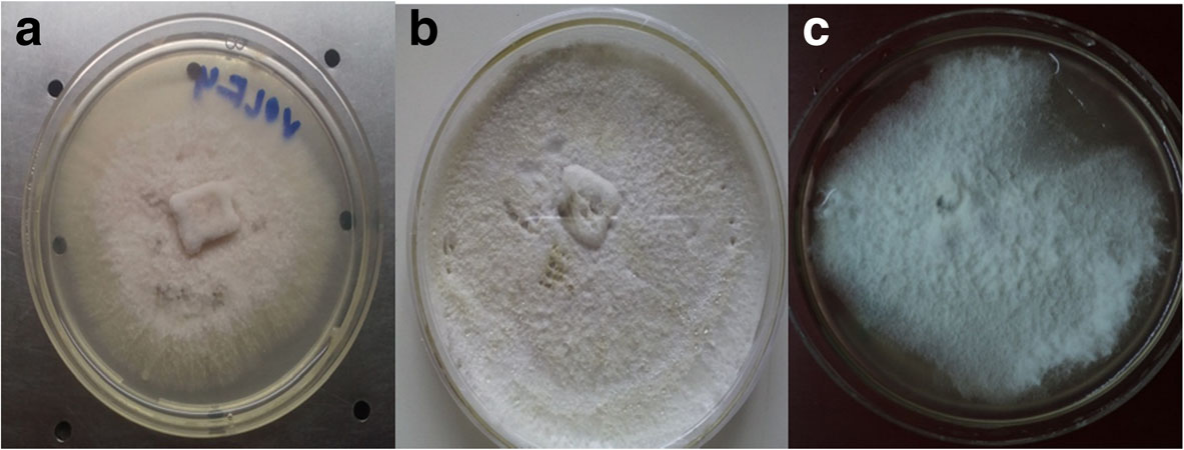
Morphological features of bioactive endophytes **a**) VOLF4 **b**) VOLF5 **c**) VOR5

**Fig. 2:**
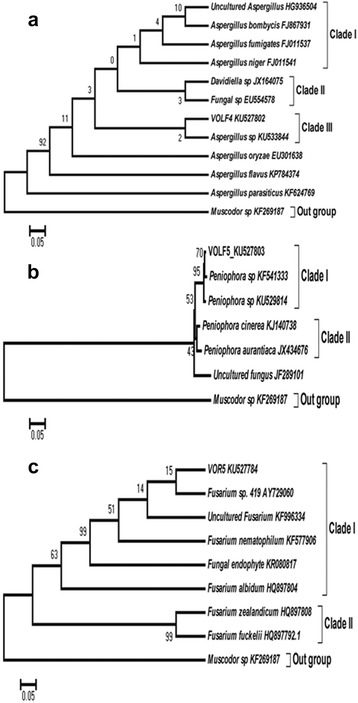
Phylogenetic tree showing rDNA based (ITS region) molecular taxonomy and phylogenetic analysis of bioactive endophytes **a**) VOLF4 **b**) VOLF5 **c**) VOR5

Morphological features of VOLF4 (*Aspergillus sp.*) VOLF5 (*Peniophora sp.*) and VOR5 (*Fusarium nematophilum*) were shown in Fig. 1. VOLF4 and VOLF5 were grown moderately over PDA medium while VOR5 was slow grower. Mycelium of VOLF4 was initially white later changed into light pink color, which is a rare color for this genus. Back was the pale in color. Rests of two endophytes were white in color. Phylogenetic positioning of these endophytes was shown in Fig. 2 suggesting unique identity of each of them.

## Discussion

Medicinal plants, which are producers of ethno-pharmacologically important secondary metabolites are the legitimate target to isolate the endophytic fungi. A number of plants already have been explored for endophytes [61–63], but this is the first report of anti-obesity potential of endophytic fungi associated with *V. odorata* collected from North Western Himalayas (Jammu and Kashmir). Few endophytes such as *F. nematophilum*, *C. trifolii, C. destructivum, C. siamense, Peniophora sp.,* and *Davidiella sp.* are reported for the first time from *V. odorata.* Out of them*, Colletotrichum trifolii* is reported first time as an endophyte.

Few antiobesity drugs such as orlistat, Qsymia are already in market and a new synthetic drug Cetilistat is under phase III clinical trials. All these drugs have some side effects [64]. Apart from these, vibralactone isolated from *Boreostereum vibrans* have also been reported to exhibit anti-obesity potential but its toxicity issues were not evaluated [65]. Thus, there is an urgent need of exploring natural resource for development of safe and effective antiobesity drugs. Endophytes are the lucrative resource of bioactive compounds, it is expected that they might have a solution of obesity also. Very few studies have been conducted on exploration of endophytic fungi for PL inhibitors [40, 41]. These studies mostly explored the endophytic broth for PL inhibitory activity. In the present study, 61% of endophytic extracts exhibited good (IC_50_ < 10 μg/mL) to moderate (IC_50_ 10–20 μg/mL) PL inhibitory activity. VOLF4 (*Aspergillus* sp*.*) extract exhibited most potent PL inhibitory activity with an IC_50_ of 3.80 μg/mL, followed by VOLF5 (*Peniophora* sp*.*) and VOR5 (*Fusarium nematophilum*) with IC_50_ of 5.85 and 6.52 μg/mL, respectively**.** Similar to our studies, Gupta et al. [40, 41] also reported good PL inhibitory activity **(**IC_50_ 3.69 and 2.12 μg/mL) of ethyl acetate extract of endophytes #57TBBALM (*Penicillium* sp*.*) and #AMLWLS (*Fusarium* sp*.*) isolated from *Aegle marmelos* and *Taxus baccata* respectively*.* Therefore, endophytes VOLF4, VOLF5 and VOR5 could be further exploited for isolating bioactive compounds, which could be used as antiobesity drugs. But some time, during fractionation and isolation of molecule, the activity was lost. Therefore in future endeavors extract of VOLF4 could be used to develop potential drug to treat obesity.

## Conclusion

The present study demonstrates that the endophytes associated with *V. odorata* have immense bioactive potential as PL inhibitors. These endophytic extracts/fractions can be explored to develop the potential drug for treating the obesity by isolating the potent molecules.

## References

[1] Strobel GA, Daisy B (2003). Bioprospecting for microbial endophytes and their natural products. Microbiol Mol Biol Rev.

[2] Clay K, Holah J (1999). Fungal endophyte symbiosis and plant diversity in successional fields. Science.

[3] Omacini M, Chaneton EJ, Ghersa CM, Müller CB (2001). Symbiotic fungal endophytes control insect host-parasite interaction webs. Nature.

[4] Brundrett MC. Understanding the roles of multifunctional mycorrhizal and endophytic fungi. In: Schulz BJE, Boyle CJC, Sieber TN. Editors. Microbial root endophytes. Springer-Verlag, Berlin, Germany; 2006. p. 281–293.

[5] Wibowo M, Prachyawarakorn V, Aree T, Wiyakrutta S, Mahidol C, Ruchirawat S, Kittakoop P (2014). Tricyclic and spirobicyclic norsesquiterpenes from the endophytic fungus *Pseudolagarobasidium acaciicola*. Eur J Org Chem.

[6] Wibowo M, Prachyawarakorn V, Aree T, Mahidol C, Ruchirawat S, Kittakoop P (2016). Cytotoxic sesquiterpenes from the endophytic fungus *Pseudolagarobasidium acaciicola*. Phytochemistry.

[7] Senadeera SPD, Wiyakrutta S, Mahidol C, Ruchirawat S, Kittakoop P (2012). A novel tricyclic polyketide and its biosynthetic precursor azaphilone derivatives from the endophytic fungus *Dothideomycete* sp. Org Biomol Chem.

[8] Strobel GA (2003). Endophytes as sources of bioactive products. Microbes Infect.

[9] Koehn FE, Carter GT (2005). The evolving role of natural products in drug discovery. Nat Rev Drug Discov.

[10] Newman DJ, Cragg GM (2007). Natural products as sources of new drugs over the last 25 years. J Nat Prod.

[11] Grundy SM (1998). Multifactorial causation of obesity: implications for prevention. Am J Clin Nutr.

[12] Khaodhiar L, McCowen KC, Blackburn GL (1999). Obesity and its comorbid conditions. Clin Cornerstone.

[13] Batterham RL, Le Roux CW, Cohen MA, Park AJ, Ellis SM, Patterson M, Frost GS, Ghatei MA, Bloom SR (2003). Pancreatic polypeptide reduces appetite and food intake in humans. J Clin Endocrinol Metab.

[14] Masaki T, Chiba S, Yoshimichi G, Yasuda T, Noguchi H, Kakuma T, Sakata T, Yoshimatsu H (2003). Neuronal histamine regulates food intake, adiposity, and uncoupling protein expression in agouti yellow (a y/a) obese mice. Endocrinology.

[15] Klok MD, Jakobsdottir S, Drent ML (2007). The role of leptin and ghrelin in the regulation of food intake and body weight in humans. Obes Rev.

[16] Millington GWM (2007). The role of proopiomelanocortin (POMC) neurones in feeding behaviour. Nutr Metab (Lond).

[17] Vicentic A, Jones DC (2007). The CART (cocaine-and amphetamine-regulated transcript) system in appetite and drug addiction. J Pharmacol Exp Ther.

[18] Shimizu H, Arima H, Watanabe M, Goto M, Banno R, Sato I, Ozaki N, Nagasaki H, Oiso Y (2008). Glucocorticoids increase neuropeptide Y and agouti-related peptide gene expression via adenosine monophosphate-activated protein kinase signaling in the arcuate nucleus of rats. Endocrinology.

[19] Xu C, He J, Jiang H, Zu L, Zhai W, Pu S, Xu G (2009). Direct effect of glucocorticoids on lipolysis in adipocytes. Mol Endocrinol.

[20] Colon-Gonzalez F, Kim GW, Lin JE, Valentino MA, Waldman SA (2013). Obesity pharmacotherapy: what is next?. Mol Asp Med.

[21] Lowe ME (1994). Pancreatic triglyceride lipase and colipase: insights into dietary fat digestion. Gastroenterology.

[22] Kokkinou M, Theodorou LG, Papamichael EM (2012). Aspects on the catalysis of lipase from porcine pancreas (type VI-s) in aqueous media: development of ion-pairs. Braz Arch Biol Technol.

[23] Van Tilbeurgh H, Egloff MP, Martinez C, Rugani N, Verger R, Cambillau C (1993). Interfacial activation of the lipase--procolipase complex by mixed micelles revealed by X-ray crystallography. Nature.

[24] Weibel EK, Hadvary P, Hochuli E, Kupfer E, Lengsfeld H (1987). Lipstatin, an inhibitor of pancreatic lipase, produced by Streptomyces toxytricini. I. Producing organism, fermentation, isolation and biological activity. J Antibiot.

[25] Christina A, Christapher V, Bhore SJ (2013). Endophytic bacteria as a source of novel antibiotics: an overview. Pharmacogn Rev.

[26] Cragg GM, Grothaus PG, Newman DJ (2009). Impact of natural products on developing new anti-cancer agents. Chem Rev.

[27] Ji HF, Li XJ, Zhang HY (2009). Natural products and drug discovery. Can thousands of years of ancient medical knowledge lead us to new and powerful drug combinations in the fight against cancer and dementia?. EMBO Rep.

[28] Challis GL (2008). Mining microbial genomes for new natural products and biosynthetic pathways. Microbiology.

[29] Dias DA, Urban S, Roessner U (2012). A historical overview of natural products in drug discovery. Meta.

[30] Clardy J, Walsh C (2004). Lessons from natural molecules. Nature.

[31] Pupo MT, Guimaraes DO, Furtado NAJC, Borges WS. Microbial natural products: a promising source of bioactive compounds. In: Taft CA. editors Modern biotechnology in medicinal chemistry and industry. Kerala, India: Research Signpost; 2006; p. 51–78.

[32] Staniek A, Woerdenbag HJ, Kayser O (2008). Endophytes: exploring biodiversity for the improvement of natural product based drug discovery. J Plant Interact.

[33] Gupta M, Saxena S, Goyal D (2014). Lipase inhibitory activity of an endophytic fungal species of *Aegle marmelos:* a bioresource for potential pancreatic lipase inhibitors. Symbiosis.

[34] Gupta M, Saxena S, Goyal D (2015). Potential pancreatic lipase inhibitory activity of an endophytic *Penicillium* species. J Enzyme Inhib Med Chem.

[35] Erhatic R, Vukobratovc M, Volf TP, Zidovec V (2010). Morphological and chemical properties of selected sweet violet populations. JCEA.

[36] Kapoor LD (1990). *V. odorata* Handbook of Ayurvedic medicinal plants.

[37] Keville K (1991). *V. odorata* Illustrated herb encyclopedia.

[38] Duke JA, Bogenschutz-Godwin MJ, Ducelliar J, Duke PAK (2002). Sweet violet (*V. odorata*). Handbook of medicinal herbs.

[39] Kermani HR, Soroush Z (2008). Effect of long-term axial spinal unloading on vertebral body height in adult thoracolumbar spine. Eur Spine J.

[40] Siddiqi HS, Mehmood MH, Rehman NU, Gilani AH (2012). Studies on the antihypertensive and antidyslipidemic activities of *V. odorata* leaves extract. Lipids Health Dis.

[41] Hamedi A, Zarshenas MM, Sohrabpour M, Zargaran A (2013). Herbal medicinal oils in traditional Persian medicine. Pharm Biol.

[42] Feyzabadi Z, Jafari F, Kamali SH, Ashayeri H, Badiee Aval S, Esfahani MM (2014). Sadeghpour O. Efficacy of *V. odorata* in treatment of chronic insomnia. Iran Red Crescent Med J.

[43] Svangård E, Göransson U, Smith D, Verma C, Backlund A, Bohlin L, Claeson P (2003). Primary and 3-D modelled structures of two cyclotides from *Viola odorata*. Phytochemistry.

[44] Khattak SG, Gilani SN, Ikram M (1985). Antipyretic studies on some indigenous Pakistani medicinal plants. J Ethnopharmacol.

[45] Koochek MH, Pipelzadeh MH, Mardani H (2003). The effectiveness of *V. odorata* in the prevention and treatment of formalin-induced lung damage in the rat. J Herbs Spices Med Plants.

[46] Svangård E, Göransson U, Hocaoglu Z, Gullbo J, Larsson R, Cleason P, Bohlin L (2004). Cytotoxic Cyclotides from *Viola tricolor*. J Nat Prod.

[47] Amer A, Mehlhorn H (2006). Repellency effect of forty-one essential oils against aedes, anopheles and culex mosquitoes. Parasitol Res.

[48] Colgrave ML, Kotze AC, Ireland DC, Wang CK, Craik DJ (2008). The anthelmintic activity of the cyclotides: natural variants with enhanced activity. Chem Bio Chem.

[49] Vishal A, Parveen K, Pooja S, Kannappan N, Kumar S. Diuretic, laxative and toxicity studies of *V. odorata* aerial parts. Pharmacol online 2009;1:739–748.

[50] Ebrahimzadeh MA, Nabavi SM, Nabavi SF, Bahramian F, Bekhradnia AR (2010). Antioxidant and free radical scavenging activity of *H. officinalis* L. *var. angustifolius*, *V. odorata*, *B. hyrcana* and *C. speciosum*. Pak J Pharm Sci.

[51] Akhbari M, Batooli H, Kashi FJ (2012). Composition of essential oil and biological activity of extracts of *V. odorata* from central Iran. Nat Prod Res.

[52] Barkatullah, Ibrar M, Ali N, Muhammad N, Meryam E (2012). *In vitro* pharmacological study and preliminary phytochemical profile of *Viola canescens* wall. Ex Roxb Afri J Pharm Pharmacol.

[53] Zarrabi M, Dalirfardouei R, Sepehrizade Z, Kermanshahi RK (2013). Comparison of the antimicrobial effects of semipurified cyclotides from Iranian *V. odorata* against some of plant and human pathogenic bacteria. J Appl Microbiol.

[54] Alireza M, Ali R (2013). Evaluation of sedative and pre-anesthetic effects of *V. odorata* Linn. Extract compared with diazepam in rats. BEPLS.

[55] Raeder U, Broda P (1985). Rapid preparation of DNA from filamentous fungi. Lett Appl Microbiol.

[56] White TJ, Bruns T, Lee S, Taylor J, Innis M, Gelfand D, Sninsky J, White T (1990). Amplification and direct sequencing of fungal ribosomal RNA genes for phylogenetics. PCR protocols: a guide to methods and applications.

[57] Altschul SF, Madden TL, Schaffer AA, Zhang J, Zhang Z, Miller W, Lipman DJ (1997). Gapped BLAST and PSI-BLAST: a new generation of protein database search programs. Nucleic Acids Res.

[58] Bustanji Y, Al-Masri IM, Mohammad M, Hudaib M, Tawaha K, Tarazi H, AlKhatib HS (2011). Pancreatic lipase inhibition activity of trilactone terpenes of *Ginkgo biloba*. J Enzyme Inhib Med Chem..

[59] Sridhar SNC, George G, Venkataramana Reddy PO, Tantak MP, Kumar D, Paul AT (2017). Synthesis, evaluation and molecular modelling studies of 2-(carbazol-3-yl)-2-oxoacetamide analogues as a new class of potential pancreatic lipase inhibitors. Bioorg Med Chem.

[60] Tamura K, Dudley J, Nei M, Kumar S (2007). MEGA4: molecular evolutionary genetics analysis (MEGA) software version 4.0. Mol Bio Evol.

[61] Katoch M, Salgotra A, Singh G (2014). Endophytic fungi found in association with *Bacopa monnieri* as resourceful producers of industrial enzymes and antimicrobial bioactive natural products. Braz Arch Biol Technol.

[62] Katoch M, Singh G, Sharma S, Gupta N, Sangwan PL, Saxena AK (2014). Cytotoxic and antimicrobial activities of endophytic fungi isolated from *Bacopa monnieri* (L.) Pennell (Scrophulariaceae). BMC Complement Altern Med.

[63] Qadri M, Johri S, Shah BA, Khajuria A, Sidiq T, Lattoo SK, Abdin MZ, Riyaz-ul-Hasan S (2013). Identification and bioactive potential of endophytic fungi isolated from selected plants of the western Himalayas. SpringerPlus.

[64] Kopelman P, Bryson A, Hickling R, Rissanen A, Rossner S, Toubro S, Valensi P (2007). Cetilistat (ATL-962), a novel lipase inhibitor: a 12-week randomized, placebo-controlled study of weight reduction in obese patients. Int J Obes.

[65] Liu D, Wang F, Liao T (2006). Vibralactone: a lipase inhibitor with an unusual fused beta-lactone produced by cultures of the basidiomycete *Boreostereum vibrans*. Org Lett.

